# Molecular pathogenic and host range determinants of reassortant Egyptian low pathogenic avian influenza H9N2 viruses from backyard chicken

**DOI:** 10.1080/23144599.2019.1637046

**Published:** 2019-07-11

**Authors:** Abdelhafez Samir, Amany Adel, Abdelsatar Arafa, Hesham Sultan, Hussein A. Hussein Ahmed

**Affiliations:** a Reference Laboratory for Veterinary Quality Control on Poultry Production, Animal Health Research Institute, Giza, Egypt; b Avian and Rabbit Diseases Dept., Faculty of Veterinary Medicine, University of Sadat, City Sadat, Minoufiya, Egypt; c Virology Dept., Faculty of Veterinary Medicine, Cairo University, Giza, Egypt

**Keywords:** LPAI H9N2, Hemagglutinin, PA, PB2, PB1, NS

## Abstract

Since the introduction of H9N2 low pathogenic avian influenza virus in Egypt, it became an endemic disease causing considerable economic losses in different poultry sectors especially in the presence of other secondary bacterial and viral infections. The H9N2 viruses in Egypt are in continuous evolution that needs deep analysis for their evolution pattern based on the genetic constitutions of the pathogenic determinant genes (HA, PB2, PB1, PA, and NS). In this work, samples were collected from the backyard chickens from 3 Egyptian governorates. Five selected viruses were sequenced and analyzed for the hemagglutinin gene which showed genetic relatedness to the Asian G1 lineage group B, similar to the circulating H9N2 viruses in Egypt since 2013. The sequence for PB2, PB1, PA, HA and NS genes of the selected five viruses indicate a natural re-assortment event with recent Eurasian subtypes and similar to Egyptian H9N2 virus isolated from pigeon in Egypt during 2014. The Egyptian viruses of our study possess amino acids signatures including S42, V127, L550, L672 and V504 in the internal genes NS1, PA, and PB2, of respectively of an impact on virus transmission and replication. This work indicates that the H9N2 is in continuous evolution with alarming to the reassortment occurrence.

## 1. Introduction

The low pathogenic avian influenza viruses of H9N2 subtype have become worldwide enzootic in the poultry population since its first detection in turkeys during 1966 [1–3] causes asymptomatic or mild disease in domestic poultry, which usually goes unnoticed. Outbreaks of H9N2 viruses have been common in chickens worldwide especially during 1994–1999 [4,5].

The H9N2 subtype virus is an extraordinary member of the influenza A group of viruses because it has a wide range of hosts including chickens, turkeys, ducks, pigs and even humans [6,7]. The low pathogenic avian influenza H9N2 viruses have adapted themselves and are circulating freely in the wild birds due to the low pathogenic nature of the their infection in natural hosts [8].

Quail and pigeon are considered as important reservoirs for the H9N2 viruses, as shown in China that they are serving as a linkage between waterfowl and terrestrial avian species [9]. In addition, both quail and porcine population act as mixing vessels for different avian influenza subtypes, that provide a suitable environment for reassortment and for creation of new viruses which may easily adapt to mammalian hosts [7,10].

The household breeding is common in the developing countries like Egypt, where the birds are raised in free range without biosecurity measures or vaccination, besides it is usually occupy a mixed rearing system. Moreover, the birds in the backyards are always in direct contact with the carrier species like the aquatic birds, and with the free flying birds like migratory birds, pigeons and quails that could be infected or carrier for different influenza subtypes [11,12]. So, household birds could play a critical role in interspecies transmission of the virus.

The avian influenza viruses are highly mutated with different ways of natural evolutionary mechanism. Reassortment is the main genetic mechanism for virus evolution and it was recorded extensively in H9N2 viruses, this mechanism occurs by acquiring the internal genes that belong the H9N2 virus from the other co-circulating subtypes like H1N1 and H3N2 that probably has a zoonotic impact on the human [13,14]. Occasionally, some reassortant H9N2 viruses in China have a significant shift in their pathogenicity in mice because they have been arisen due to the co-circulation with high pathogenic avian influenza viruses like H5N1 [15]. The Egyptian low pathogenic H9N2 viruses elicited a continuous evolution either in the level of HA gene sequence or the internal genes. In recent studies, some Egyptian viruses from quails have genetically and antigenically evolved [16,17]. In addition to the reporting of two viruses isolated from pigeon which have earned internal genes from Eurasian avian influenza viruses that have circulated in wild birds. There are some mutations were detected in the internal genes with evidence of reassortment, which probably elevates the ability of virus transmission and pathogenicity in mammals [17].

This study targeted the viruses from the backyard chicken as this sector has the most contact to pigeons and wild birds which consequently facilitate exposure to the human. As well as genomic profiling of the pathogenic determinant genes of H9N2 viruses has also been examined to investigate the evolutionary dynamics of H9N2 viruses in Egypt.

## 2. Material and methods

### 2.1. Samples

Oropharyngeal swabs were collected from five suspected backyard chicken populations suffered from respiratory disorder, nasal and ocular discharge, from 3 Egyptian governorates (Table S1). Sampling was done during the first half of 2015 (from January till June), then the gathered samples were pooled, isolated and examined in the Reference Laboratory of Veterinary Quality Control on Poultry Production in Egypt (RLQP).

### 2.2. Detection of the H9N2 virus by real-time RT-PCR

Viral RNAs were extracted from the targeted samples using the QIAamp Viral RNA Mini Kit (Qiagen, Hilden, Germany) following its work instructions. The targeted samples were examined using one-step real-time RT-PCR (Qiagen, Hilden, Germany) for the M gene of influenza type A viruses [18] using the real-time PCR Mx3000P System (Agilent, California, USA). Positive AIV RNAs were subtyped as H9 subtype using specific primers [19], Thermal profile for amplification of HA gene of H9 subtype was done as follows: 50°C for 30 min, 95°C for 15 min, cycling steps of 94°C for 10 s, 54°C for 30 s and 72°C for 10 s repeated for 40 cycles.

### 2.3. Virus propagation

The positive five samples were propagated in 9–11 day old SPF ECE by intra-allantoic inoculation, then incubated at 37°C for 3–5 days. The allantoic fluids were harvested, and tested for HA activity according to OIE manual 2015, then they were retested for the H9 subtype for isolate confirmation by specific real-time RT-PCR as described before [19].

### 2.4. Genomic profiling of the pathogenic determinant genes of H9N2 viruses

#### 2.4.1. Amplification of viral genes

The selected 5 isolates were replicated for Hemagglutinin, PB2, PB1, PA, and NS genes by real-time RT-PCR using Qiagen one step RT-PCR® kit (QIAGEN, Hilden, Germany) with primers in Table S2. The real-time RT-PCR cycling ran as one cycle of reverse transcriptase step for 30 min at 50°C, then one cycle for 15 min at 95°C for initial denaturation followed by 40 cycles of 94°C for 45 sec, 56°C for 45 sec and 72°C for 2 min and final extension for 10 min at 72°C. The RT-PCR reactions were thermally incubated in Applied Biosystems thermocycler, USA. Then, The RT-PCR products were electrophoresed on a stained agarose gel 1.5% with Ethidium bromide dye, the expected positive products were visualized at their defined molecular weights against the DNA marker on gel documentation system – Image capture (Biometra, Germany).

#### 2.4.2. Genome sequencing

The amplicons of the different genes (HA, PB2, PB1, PA, and NS) were purified by Qiaquick kit, then sequenced using 2 μl of Big Dye Terminator V3.1 cycle sequencing kit (Perkin-Elmer, Foster City, CA), 1 μl of each specific primer for each gene per reaction as shown in Table S2. The thermal cycling protocol for sequence reactions was executed as follows: one cycle at 96°C for 1 min, 25 replication cycles of 96°C for 10 Sec, 50°C for 5 Sec and 60°C for 2 min. Then, a spin column Centrisep® kit (Applied Biosystems, USA) was used in purification of the sequencing reactions to remove the excessive free dNTPs. After cleaning up, the purified sequence reactions were loaded in a sequencer plate of Applied Biosystems (ABI 3130 genetic analyzers, USA). The output sequences of this study were designated and submitted to the Genebank of the national center of biotechnology information, USA (NCBI) with accession numbers as listed in Table S3.

#### 2.4.3. Mutational and phylogenetic analysis

The sequenced genes of this study were BLAST in National Center Bank for Information NCBI (https://www.ncbi.nlm.nih.gov/), the representative sequences of H9N2 viruses that genetically related to our viruses used in this study have been retrieved from the NCBI database. Version 7.1 of Bioedit software [20] was employed for the mutation analysis by creating the comparable alignment for nucleotide and amino acid sequences, also it was used for calculating the identity matrix between the different sequences that were inclusive in this study. Mega7 software was utilized in constructing the phylogenetic tree [21], the compatible method for the evolutionary study is maximum likelihood (ML) tree method was applied [21], with general time-reversible (GTR) is the model of nucleotide substitution that was used with gamma-distributed rate variation among sites of estimated proportion of invariant sites (I) (with 8 rate categories, Γ4). Bootstrap analysis was accomplished with 1000 replicates. NetNGlyc 1.0 Server is an open access server that is capable of determination of the glycosylation sites on the hemagglutinin gene [22].

### 2.5. Evolutionary rates and divergence times of the Egyptian H9N2 viruses

This analysis was carried out with the program package BEAST version 1.8.4 [23], the approximate time of most recent common ancestor (tMRCA) for each gene was estimated in alignments including our sequences and other genetically related sequences with defined dates of collection. The analysis was done at 100 million Monte Carlo Markov Chain (MCMC) generations with HKY (Hasegawa–Kishino–Yano) substitution model; with the lognormal uncorrelated relaxed clock – gamma heterogeneity model [24,25]. The evaluative Effective sample size (ESS) was achieved by Tracer v1.6 for each individual run when the ESS for molecular clock parameters were over 200, the data were engaged in the analysis [26]. The estimated lower and upper extremities of the 95% highest posterior density interval (HPD) were indicative of the intensity of the data distribution. Maximum clade credibility (MCC) trees were constructed after a 10% burn-in removal by using TreeAnnotator v1.8.4 (http://beast.bio.ed.ac.uk/TreeAnnotator) and the MCC trees were shown with FigTree v1.4.3 [27] (http://beast.bio.ed.ac.uk/figtree).

## 3. Results

### 3.1. Real-time RT-PCR for H9 detection and virus isolation

The 5 samples were positive for H9 subtype by real-time RT-PCR with (ct) values ranged from 13.22 to 20.22 as listed in Table S1. The positive samples were propagated and then examined for HA activity and the collected allantoic fluids have HA unites between 2log7-2log9. The isolates were confirmed as H9 subtype by real-time RT-PCR.

### 3.2. Genetic analysis of the sequenced genes

#### 3.2.1. Hemagglutinin (HA)

**Figure 1.**
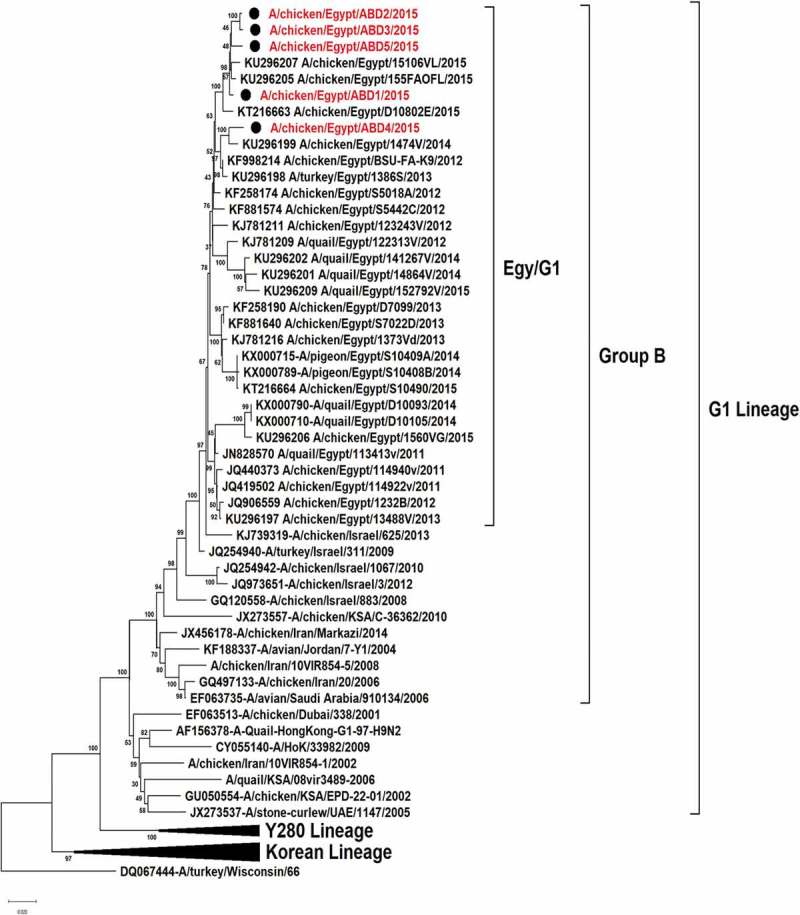
Phylogenetic tree of HA gene for 49 selected Egyptian viruses; the red labeled taxa are the 5 viruses of this study.

All the 5 viruses in this study were sequenced for the hemagglutinin gene that was genetically related to the circulating Egyptian H9N2 viruses that persist from 2013, with high similarity to the circulating viruses in 2014 and 2015 with identity 98–99%. Phylogenetically, all the Egyptian viruses have been related to the group B of the G1 Lineage, with multiple minor subgroups (Figure 1)

The analysis of the glycosylation sites on HA sequences revealed 6 potential sites (**^11^**NSTE**^14, 87^**NGTC**^90,123^**NVTY**^126, 280^**NSTL**^283, 287^**NISK**^290^**^,^ and **^474^**NGTY**^477^**) as shown in all circulating Egyptian viruses without any acquisition for new sites in comparison to the reference strain of G1lineage (A-Quail-HongKong-G1-97).

The Hemagglutinin proteins of the viruses in this study have mammalian-like receptors T125, L216, H173 and K228 as shown in the (Table 1). The comparison between the receptor binding sites of the 5 Egyptian viruses in this study revealed a substitution A180V in one virus (A/chicken/Egypt/ABD3/2015). The cleavage site motif of these isolates is typically like the low pathogenic avian influenza motif **^315^-**PARSSRGLF-**^323^** which does not have multiple basic amino acids (Table 2).

**Table 1.** Host range genetic determinants in H9N2 viruses isolated from backyard chicken in Egypt.

gene	site	Avian markers	Mammalian markers	Viruses of the study
HA	125	A	T	T
	216	Q	L	L, Q
	228	R	K	K
	173	Q	H	H
	180	A	E	V
PB2	318	K	R	R
PA	382	E	D	D
NS1	227	E	K/R	E/K

**Table 2.** The Receptor binding sites and cleavage sites on the HA of the five H9N2 isolates of this study.

				RBS		
	cleavage sites 315–323 (324–332)	Right edge of RBS 128–132 (134–138)	Left edge of RBS 214–219 (224–229)	145	180	173
**A/quail/Egypt/113413v/2011**	PARSSRGLF	GTSKS	NGLIGR	**T**	**A**	**H**
**A/Chicken/Egypt/ABD1/2015**	PARSSRGLF	GTSKS	NGLIGR	**T**	**A**	**H**
**A/Chicken/Egypt/ABD2/2015**	PARSSRGLF	GTSKS	NGLIGR	**T**	**A**	**H**
**A/Chicken/Egypt/ABD3/2015**	PARSSRGLF	GTSKS	NGLIGR	**T**	**V**	**H**
**A/Chicken/Egypt/ABD4/2015**	PARSSRGLF	GTSKS	NGLIGR	**T**	**A**	**H**
**A/Chicken/Egypt/ABD5/2015**	PARSSRGLF	GTSKS	NGLIGR	**T**	**A**	**H**

The cleavage site of the targeted viruses is typically for the low pathogenic H9N2 viruses. Also all the receptor binding sites are the same like in the first introduced Egyptian strain in 2011, except A/Chicken/Egypt/ABD3/2015 that reveals one substitution A180V.

The antigenic sites that characterize the H9 epitope mapping have no alteration in comparison with the first recorded Egyptian isolate (quail/3134V/2011), especially D135, N183 and L216 in antigenic site II; and S127, T179 and D189 in overlapping antigenic site.

#### 3.2.2. Polymerase basic protein2 (PB2)

**Figure 2.**
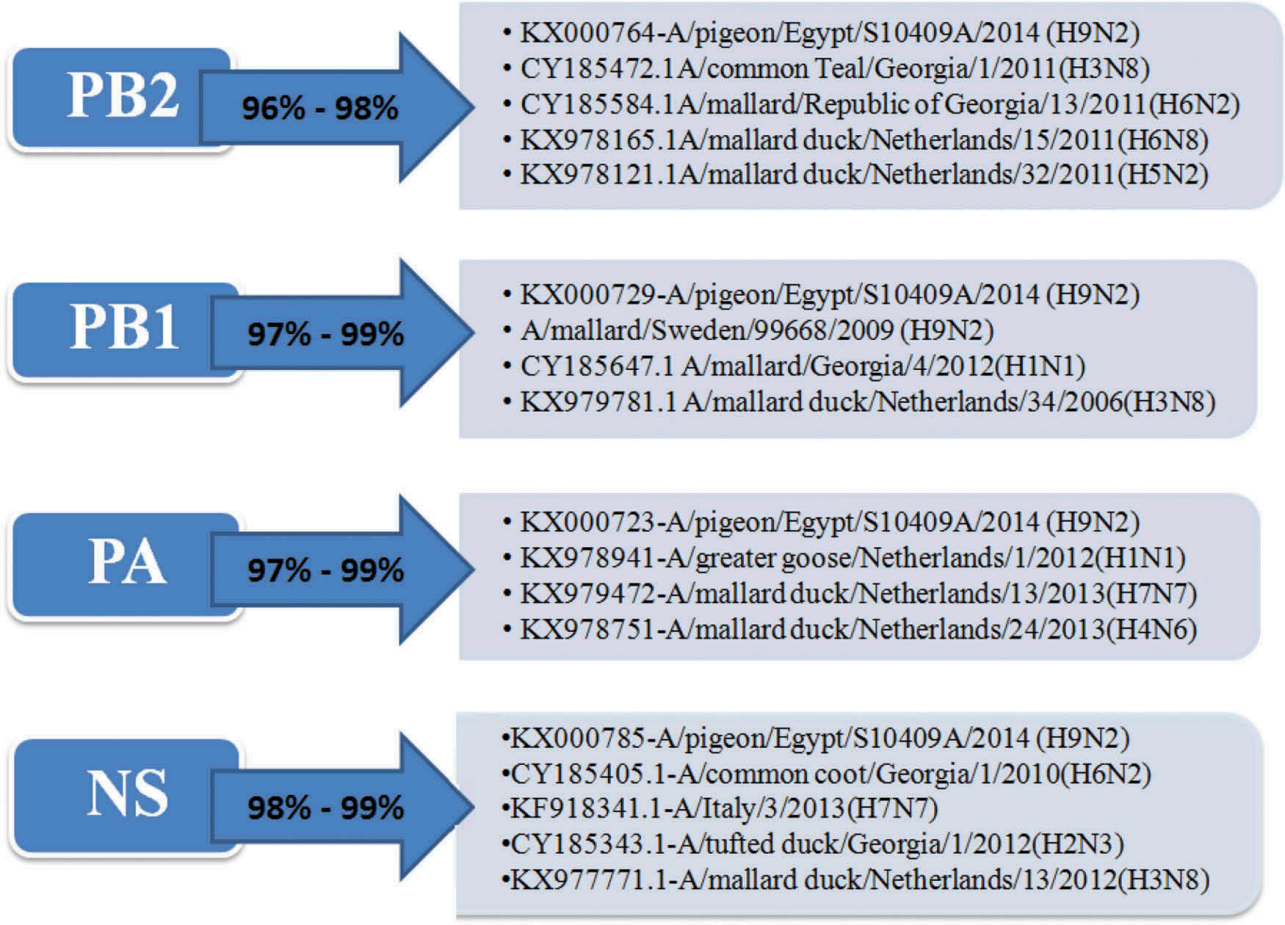
Similarity of the Egyptian reassortant H9N2 viruses with their genetically related viruses.

**Figure 3.**
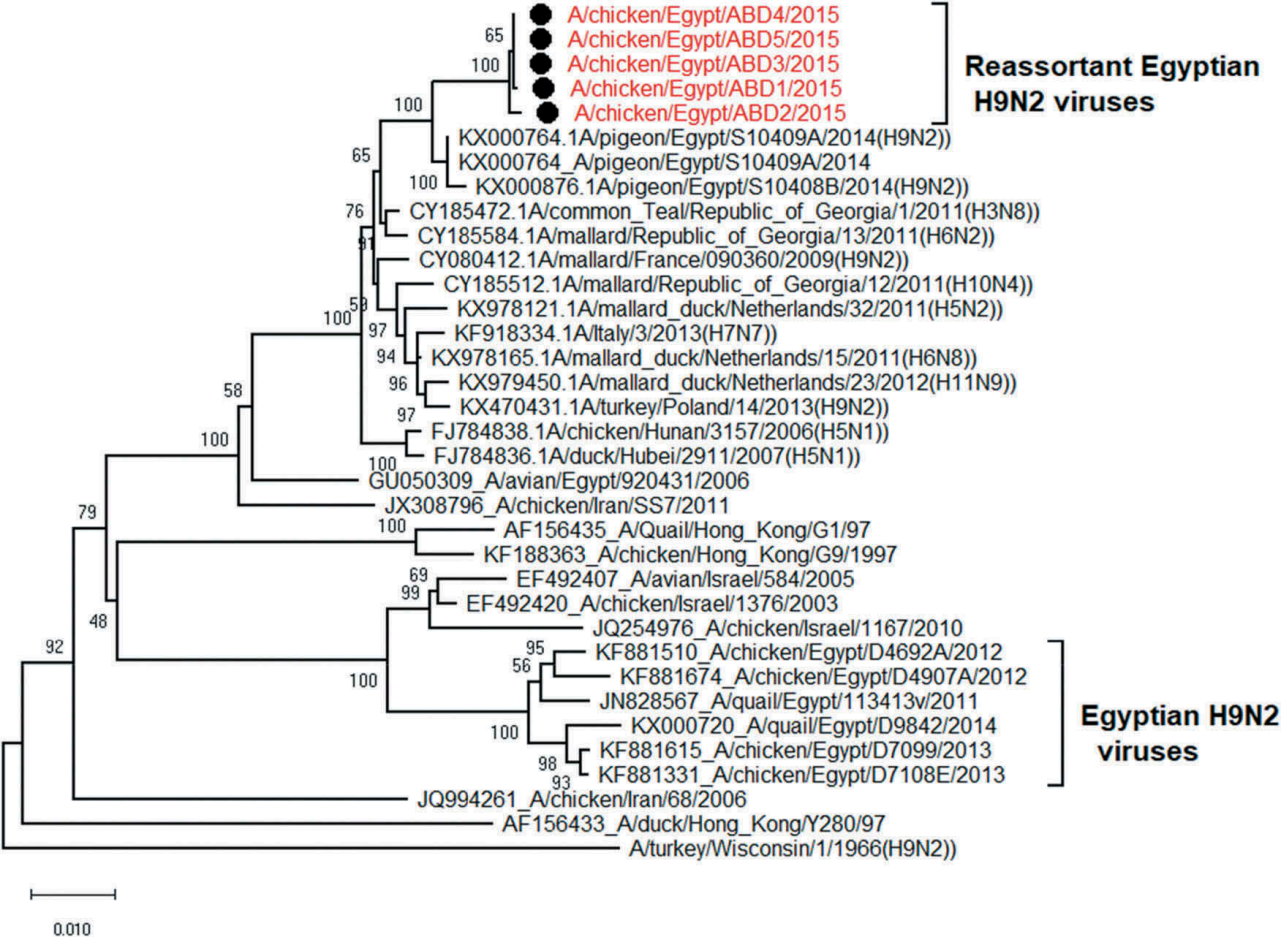
Phylogenetic tree of PB2 gene for 5 selected Egyptian viruses labeled with red taxa, showed highly genetic relatedness with A/pigeon/Egypt/S10409A/2014, A/mallard/Republic of Georgia/13/2011(H6N2) and A/common Teal/Georgia/1/2011(H3N8).

The PB2 gene of the 5 selected isolates has no genetic relationship with that of the circulating Egyptian H9N2 viruses and all the G1 lineage viruses except the Egyptian virus that isolated from a pigeon in 2014 (A/pigeon/Egypt/S10409A/2014) which is the most identical to the viruses of our study with similarity 99%. Also they have high similarity with other low pathogenic avian influenza with identity 96%-97%- with A/mallard/Republic of Georgia/13/2011(H6N2) and A/mallard duck/Netherlands/32/2011(H5N2) and A/common Teal/Georgia/1/2011(H3N8) as shown in (Figures 2 and 3)

There is a substitution K318R that related to the mammalian influenza viruses (Table 1) and anther substitution I504V which is characteristic for the highly pathogenic viruses (Table 3). There are no alterations in amino acid residues Q591, E627, and D701 which are characteristic for the low pathogenic avian influenza viruses.

**Table 3.** Pathogenic determinants in the 5 Egyptian H9N2 viruses isolated from backyard chicken.

gene	site	High Pathogenic	Low pathogenic	Viruses of the study	Egyptian H9N2 (qu/v3413/2011)
PB2	504	V	I	V	I
PA	127	V	I	V	V
	672	L	F	L	L
	550	L	I	L	L
NS1	42	S	A/P	S	S
	PDZ domain-binding motif	ESEV	KSEV	ESEV KSEV	KSEV

#### 3.2.3. Polymerase basic protein1 (PB1)

**Figure 4.**
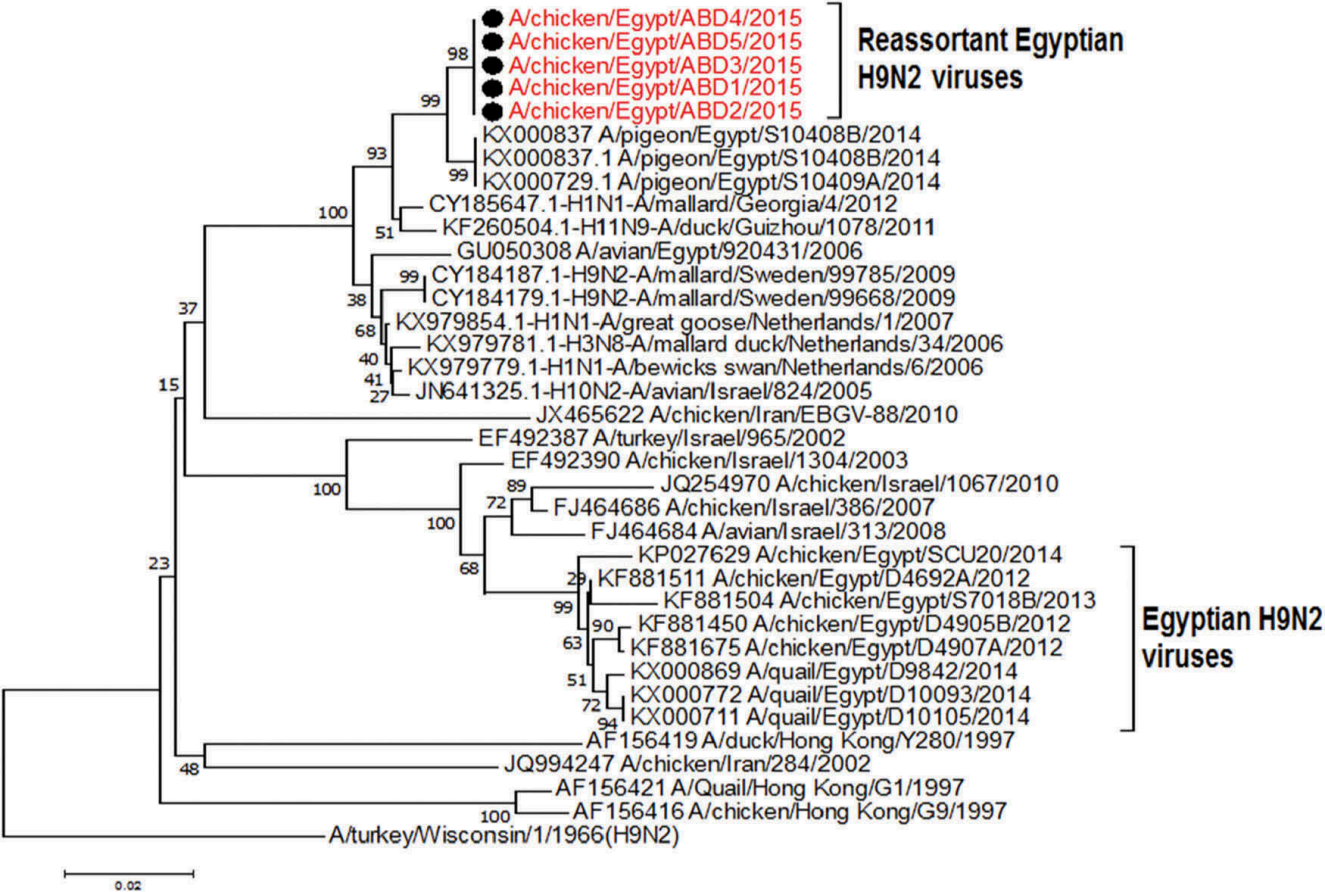
Phylogenetic tree of PB1 gene for 5 selected Egyptian viruses labeled with red taxa showed highly genetic relatedness with A/pigeon/Egypt/S10409A/2014 and H1N1 virus from Georgia in 2012 and the H9N2 viruses from Sweden in 2009.

The partial sequences for the PB1 gene of the selected 5 viruses are closely identical to the Egyptian virus from pigeon (A/pigeon/Egypt/S10409A/2014) with identity 99%, that are genetically related to the A/mallard/Georgia/4/2012(H1N1), A/mallard duck/Netherlands/34/2006(H3N8) and A/mallard/Sweden/99,668/2009 (H9N2) with identity% 97–98% . Those were clarified in (Figures 2 and 4)

#### 3.2.4. Polymerase acidic protein (PA)

**Figure 5.**
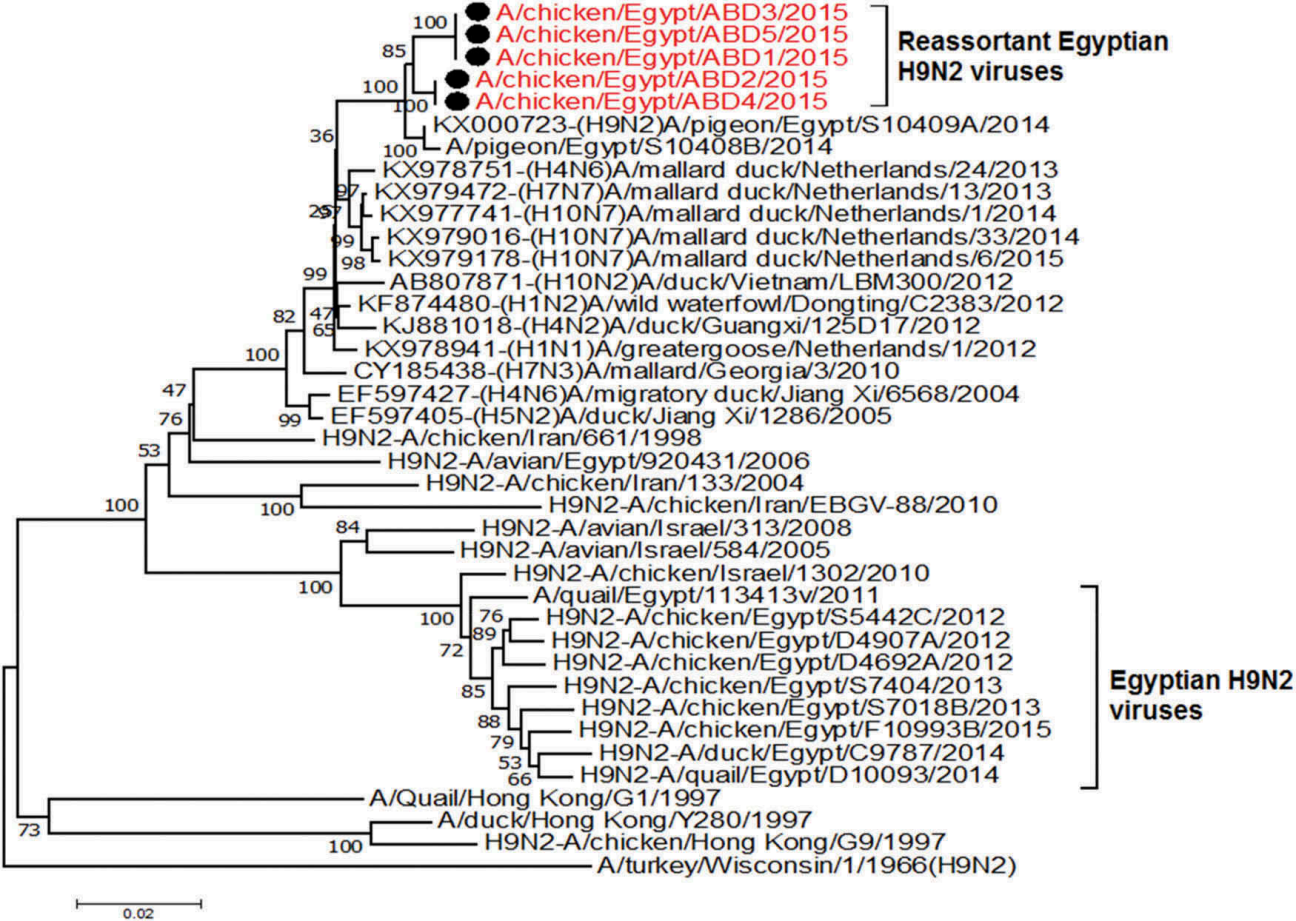
Phylogenetic tree of PA gene for 5 selected Egyptian viruses labeled with red taxa, showed highly genetic relatedness with A/pigeon/Egypt/S10409A/2014, A/mallard duck/Netherlands/24/2013(H4N6) and A/mallard duck/Netherlands/13/2013(H7N7).

The PA gene of the 5 viruses is highly similar (99%) to the Egyptian pigeon’s virus (A/pigeon/Egypt/S10409A/2014) which are genetically closely related to A/greater goose/Netherlands/1/2012(H1N1), A/mallard duck/Netherlands/13/2013(H7N7) and A/mallard duck/Netherlands/24/2013(H4N6) with identity % 98% as shown in (Figures 2 and 5)

**Figure 6.**
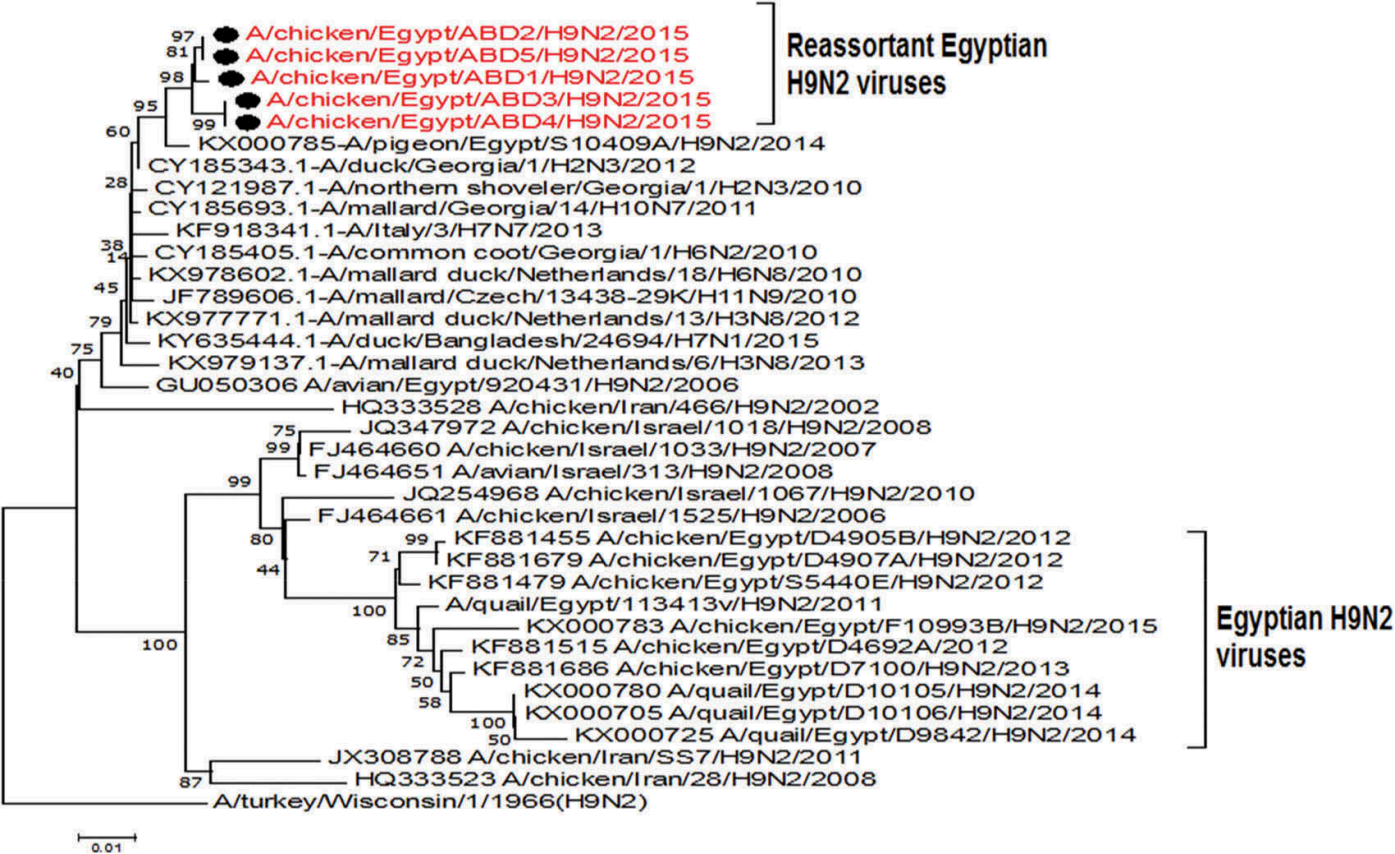
Phylogenetic tree of NS gene for 5 selected Egyptian viruses labeled with red taxa, showed highly genetic relatedness with A/pigeon/Egypt/S10409A/2014, A/Italy/3/2013(H7N7) and A/common coot/Georgia/1/2010(H6N2).

A substitution E382D which is one of the mammalian host specific marker (Table 1), in addition to amino acids substitutions V127, L672, and L550 that are associated with virulence wereobserved in the targeted viruses (Table 3).***3.2.5 NS***:

The NS gene of the studied viruses also has the highest identity (99%) with the (A/pigeon/Egypt/S10409A/2014), that are highly similar and genetically related to A/common coot/Georgia/1/2010(H6N2), A/Italy/3/2013(H7N7) and A/mallard duck/Netherlands/13/2012(H3N8) with identity % 98–98.5% that shown in (Figure 2) and the phylogenetic tree was shown in (Figure 6). There is no alteration in the pathogenic determinants on NS1 except at amino acid residue S42 which is considered as a marker for highly pathogenic avian influenza (Table 3) and E 227 which is one of the mammalian markers (Table 1).

### 3.3. Evolutionary rate and temporal analysis

The selected viruses were analyzed for the time of the most recent common ancestor (tMRCA) for each gene that showed presumptive evidences for re-assortment with other different avian influenza subtypes. As shown in the (Table 4), the estimated time of the common ancestor of NS gene with re-assortment was emanated at the end of 2012 from different subtypes circulated in Georgia and Netherlands during 2010–2012, with evolution rate 3.7*10^−3^ and 95% HPD = 2.3–5.13*10^−3^. PB1 gene also has a common ancestor from viruses of different subtypes circulated in Georgia and Netherlands during 2010–2012 and with reassortant H9N2 virus from Sweden (A/mallard/Sweden/99,668/2009) with evolution rate 5.11*10^−3^ and 95% HPD = 3.49–6.9*10^−3^. Furthermore, the evolution rate of PA gene is 4.5 *10^−3^ and 95% HPD = 3.38–5.8*10^−3^ that have a presumptive evidence for genetic re-assortment with H7N7, H1N1 and H4N6 from the Netherlands with an estimated time of the common ancestor since early 2013. Finally, the reassortant PB2 gene of the studied isolates have a genetic lineage to H6N2 and H5N2 from Georgia and Netherlands with an estimated time of the common ancestor since the middle of 2012, the PB2 genes have evolution rate = 4.58 *10^−3^ and 95% HPD = 3.31 − 5.96*10^−3^.

**Table 4.** Estimated evolutionary rates of nucleotide substitutions per site per year (subs/site/year) for H9N2 viruses circulating in Egypt between 2011 and 2015.

	ULc – skyline Substitution rates (subs/site/year)		Time of the most recent common ancestor tMRCA (decimal date)	
Genome segment	Mean (*10^−3^)	95% HPD (*10^−3^)	Reassortant group	Common group
NS	3.7	2.3–5.13	2012.89	2009.4
PA	4.5	3.38–5.8	2013.08	2009.4
PB1	5.11	3.49–6.9	2012.86	2010.6
PB2	4.58	3.31–5.96	2012.5	2009
HA	5.7	4.4–7.09	2013.9	2009

Substitution rates were estimated under an uncorrelated lognormal relaxed clock (UL) model. The 95% HPD intervals are given

## 4. Discussion

The low pathogenic avian influenza virus H9N2 usually induces mild asymptomatic infections that are mostly undetected. This situation allows the virus to be evolved via adaptive mutations and induces an enzootic widespread infection. Another way for virus evoulation is through genetic reassortments in its genomic segments with other avian influenza subtypes for instance with H1N1, H5N1and H7N7 subtypes. Thereby new variant strains or new reassortant viruses have been emerged and that will lead to change in the biological properties of the new virus [28,29]. There are many factors that make H9N2 viruses an excellent candidate for the next influenza pandemic such as their wide host range, their ability to cross the species barrier, ecological diversity, antiviral resistance and finally their zoonotic importance. Beside many other factors such as ineffective vaccination, immunological pressures and limited surveillance activity that contribute to its persistence and evolution [28].

In the present study, a total number of 5 suspected backyard flocks were sampled and collected from chicken from 3 governorates in Lower Egypt (Delta region), these flocks showed mild clinical signs as ruffled feathers, depression, and respiratory distress. Then, the 5 collected samples were positive for low pathogenic avian influenza H9N2 by real-time RT-PCR.

In previous studies on the Egyptian H9N2 viruses, the HA gene is shown to be genetically related to the G1 lineage in the evolutionary group B [30] and classified phylogenetically as one major group with multiple minor clusters. In addition to a newly emerged variant cluster that was evolved since 2012 and recorded up to 2015 from quails [16,17]. Thereby, the 5 sequenced hemagglutinin gene of this study were phylogenetically located in one group with the Egyptian viruses circulating since 2013.

The main pathogenic determinant of influenza viruses is the proteolytic cleavage site on HA. In this study, all isolates have the typical motif of low pathogenic avian influenza virus (PARSSR/GLF) [31]. This motif has a single Arginine (R) residue which is recognized by trypsin-like proteases secreted mainly in respiratory organs and intestine and that is the reason why H9N2 virus infection localized in these organs [31].

All Egyptian H9N2 viruses have conserved receptor binding domains at amino acid residues Y91, W143, T145, L184 and Y185 (corresponding to 98, 153, 155, 194 and 195 respectively in H3 numbering) [32]. However, the Egyptian viruses isolated from chicken in this study have H173 in the receptor binding pockets and L216 (226 H3 numbering) in the Left-edge of the RBS on the HA1 which are specific for human viruses [33]. All Egyptian chicken viruses in this study contain amino acid residues H173, T179 and L216 (183, 189 and 226 H3 numbering) in the HA1. Those residues supposed to sustain the binding to mammalian α2,6-sialic acid-linked receptors and the replication in the human respiratory epithelial cells [14,34]. The HA of the targeted viruses of this study has potential 6 glycosylation sites like the motifs of all the Egyptian viruses including in Egy/G1 [16], including **^11^**NSTE**^14, 87^**NGTC**^90, 123^**NVTY**^126, 280^**NSTL**^283, 287^**NISK**^290^**, and **^474^**NGTY**^477^**, without acquisition of any additional glycosylation sites.

The I504V substitution in the PB2 gene is associated with enhanced activity of the polymerase complex [35]. This mutation was associated with the mammalian-specific determinant K318R in the sequences of this study. However, the two substitutions (E627K and D701N) that were associated with virulence and virus transmission to mammals were not observed [36,37]

The deduced PA amino acid sequences of our viruses had a mammalian host specific residue, E382D, which present in all PA of previously known Egyptian H9N2 viruses [17]. In addition to three substitutions V127, L672, and L550 that are associated with virulence were also observed in the Egyptian H9N2 viruses.

PA-X is an additional virus protein that composed of the first 191 N-terminal amino acids identical to PA with a completely different C-terminal domain of 61 amino acids that found due to a ribosomal frameshifting [38]. PA-X protein plays an important role in inhibiting cellular protein synthesis [39]. The absence of PA-X in H9N2 virus decreased its replication and pro-inflammatory response in mice, on the contrary to the positive impact of its absence on H1N1 and highly pathogenic H5N1 viruses. So, the function of PA-X could be virus strain-specific [39]. Deletion of 20 amino acids in the C-terminus of PA-X has severely reduced nuclease activity [38]. All amino acid sequences of the PA-X in our targeted viruses and other Egyptian viruses in general are intact without deletion.

NS1 protein is critical for natural infection due to its ability to inhibit the host innate immune response [40] through species-specific interactions with multiple host proteins [41]. NS1 has been studied extensively as a molecular determinant of virulence. Influenza viruses lacking the IFN antagonist NS1 are only able to replicate in cells or mice that have a compromised IFN response [42]. This effect requires glutamic acid (E) at amino acid position 92 of the NS1 molecule and allows virus replication in the presence of IFN; this mutation was a determinant of virulence in pigs. Furthermore, E92 increases the virulence of HPAI H5N1 in mice [43].

The H9N2 viruses in this study had PDZ motif (X-S/T-X-V) at C-terminal of NS1 protein in the avian form of ESEV in 3 viruses A/Chicken/Egypt/ABD1/2015, A/Chicken/Egypt/ABD2/2015, and A/Chicken/Egypt/ABD5/2015 while the remaining 2 viruses had KSEV motif. Previously, it has been found that avian ESEV motif of the NS1 protein of the H1N1 or H7N1 could enhance the ability to bind with more mammalian PDZ domain proteins [44]. The NS1 of the H9N2 viruses harbored the mammalian-specific E227K substitution. Interestingly, the NS1 of viruses has S42 which is previously known as a virulence determinant in mammalian host especially for H5N1 viruses [45].

Both PB2 and NS genes have a common ancestor of H6N2 that circulated in Georgia during 2010–2012. Furthermore, NS gene was genetically related to the highly pathogenic avian influenza H7N7 from Italy 2013 which were isolated from 3 human cases in contact with infected chicken [46].

Based on the temporal analysis for evolution of the selected viruses that was shown in (Table 4), the evolution time of the most recent common ancestors (TMRCA) of the reassortant viruses was estimated in the middle of 2012 to late 2013 originated from the Eurasian avian influenza subtypes for the re-assorted segments (PB2, PB1, PA and NS). The viruses have the highest nucleotide substitution rate for the HA gene segment (5.7*10^−3^) followed by PB2 and PA genes (4.58*10^−3^ and 4.5*10^−3^, respectively). Thus, these findings indicate the rapid evolution of the H9N2 viruses in Egypt with high risk of re-assortment with the co-circulating subtypes.

From all the above, this study elicited 5 H9N2 viruses inherited 4 internal genes from Eurasian AIVs circulating in wild birds and pigeon in Egypt [17]. Pigeons act as a bridge species because of their crucial role in the transition of avian influenza viruses between poultry and migratory birds. Evolution of PB2, PB1, PA, NP and NS genes of the Egyptian H9N2 viruses of this study that are genetically related to other Eurasian avian influenza subtypes like H1N1, H7N7, H6N2, and H5N3, indicates occurrence of multiple re-assortments in migratory birds while their emigration from Asia and Europe to the Middle East countries [17]. Thus, these reassortant viruses could be transmitted from the migratory birds and pigeon to the contact backyard chickens. Besides, there is a possibility for re-assortment of H9N2 with the highly pathogenic H5N1 as both of them are endemic and circulating together in different mammalian and avian hosts in Egypt, as the re-assortment was done in vitro resulted in reassortant viruses with a wide range of pathogenicity [47].

## 5. Conclusion

The low pathogenic H9N2 viruses in Egypt still in persistent evolution and emergence of multiple reassortant viruses originated previously from pigeons and recorded here in backyard chickens. Thereby, the emergence of re-assortant strains could be the gate for evolution of new influenza subtypes and it may increase the mammalian susceptibility and the zoonotic impact on the contact humans. Therefore, it is recommended to conduct further studies on the transmission and pathogenicity of these reassortant viruses. This should go parallel to the effective survey targeting all poultry sectors and bird species to recognize the host range, transmission, and geographic distribution of these reassortant viruses.
