# Supplementary material for: Molecular pathogenic and host range determinants of reassortant Egyptian low pathogenic avian influenza H9N2 viruses from backyard chicken
Source: Int J Vet Sci Med. 2019 Jul 11;7(1):10–9. doi: 10.1080/23144599.2019.1637046 (PMC6776986; doi:10.1080/23144599.2019.1637046)
Supplement: Supplemental Material [file TVSM_A_1637046_SM9960.zip › Supplementary table 1.doc]

**Supplementary table 1: Epidemiological data of the collected samples**

| **No.** | **Isolate ID** | **Governorate** | **Type of production** | **Ct** | **HA titer** |
| --- | --- | --- | --- | --- | --- |
| **1** | A/Chicken/Egypt/ABD1/2015 | Gharbia | backyard | 20.18 | 6 |
| **2** | A/Chicken/Egypt/ABD2/2015 | Monofia | backyard | 15.31 | 4 |
| **3** | A/Chicken/Egypt/ABD3/2015 | Qalyobeya | backyard | 18.98 | 8 |
| **4** | A/Chicken/Egypt/ABD4/2015 | Gharbia | backyard | 20.22 | 8 |
| **5** | A/Chicken/Egypt/ABD5/2015 | Gharbia | backyard | 20.22 | 8 |
