# Supplementary material for: Molecular pathogenic and host range determinants of reassortant Egyptian low pathogenic avian influenza H9N2 viruses from backyard chicken
Source: Int J Vet Sci Med. 2019 Jul 11;7(1):10–9. doi: 10.1080/23144599.2019.1637046 (PMC6776986; doi:10.1080/23144599.2019.1637046)
Supplement: Supplemental Material [file TVSM_A_1637046_SM9960.zip › Supplementary table 2.doc]

Supplementary table 2: the primers sets used in the amplification and sequence of the PB2, PB1, PA, HA and NS genes:

| **Primer ID** | **Nucleotide Sequence** | **Length** | **Reference** |
| --- | --- | --- | --- |
| **PB2 Primers** | | | |
| **PB2-1FV2** | 5'GGAAAGCAGGTCAAATATATTCAATA 3' | 26 | **FLI** |
| **PB2-F-503** | 5’ATGGAGGTCGTTTTCCCAAATGAGG 3’ | 25 | **RLQP** |
| **PB2-F910** | 5’TGAGGAACAAGCTGTGGATAT3’ | 21 | **SEPRL** |
| **PB2-F1361** | 5’ATTGATAATGTCATGGGGATG 3’ | 21 | **RLQP** |
| **PB2-1421R** | 5’ GGT AAT AAT CCG ATC ATC CCC AT 3’ | 23 | **RLQP** |
| **PB2-F-1775** | 5'TAG TAC CAA AGG CTG CCA GA 3' | 20 | **RLQP** |
| **PB2-1RV2** | 5'GGTCGTTTTTAAACAATTCGACAC 3' | 24 | **FLI** |
| **PB2-R397** | 5'ACTTGGTTTCGGAAATGAAC3' | 20 | **RLQP** |
| **PB2-R828** | 5'TCTCCAGCAGTGATGCCAGTG 3' | 21 | **RLQP** |
| **PB2-R-2317** | 5'CTA TTC GAC ACT AAT TGA TGG C 3' | 22 | **RLQP** |
| **PB1 Primers** | | | |
| **PB1-F-1433** | 5’GCA AGC TAG TCG GAA TCA A 3’ | 19 | **RLQP** |
| **PB1-R432** | 5’AATGTCCAGTCATAGGT CTG 3’ | 20 | **RLQP** |
| **Pb1 R-867** | 5’CAC GAC GTT TGC CAA TTT AGC C 3’ | 22 | **RLQP** |
| **PB1-1230R** | 5’ATGTTGAACATGCCCATCATCAT3’ | 23 | **RLQP** |
| **PB1-R-2322** | 5’TTC ATG AAG GAC AAG CTA A 3’ |  | **RLQP** |
| **PA Primers** | | | |
| **PA -3FV2** | 5'AGC GAA AGC AGG TAC TGA TTC AAA A 3' | 25 | **FLI** |
| **PA-F-450** | 5'CAC AGG GGA GGA AAT GGC AAC C3' | 22 | **RLQP** |
| **PA+734** | 5'AACCGAACGGCTGCATTGA 3' | 19 | **RLQP** |
| **PA-1131F** | 5'ACT CGG TGA GAA CAT GGC ACC3' | 21 | **RLQP** |
| **PA-F1825** | 5'ACATGACCAAAGAATTCTTTGA3' | 22 | **RLQP** |
| **PA-R-510** | 5'GCC CTG CTC TCT TCA TCA AGG G3' | 22 | **RLQP** |
| **PA-R-960** | 5'ATGTTGGGCTCCTTCCACCCGAAAAATG3 | 28 | **RLQP** |
| **PA -1312R** | 5'CTC CTA TTT CAT CAA GTT CTA TCC 3' | 24 | **RLQP** |
| **PA-R-1621** | 5'CTT TTC CCA CTT GTG TGG CTC C3' | 22 | **RLQP** |
| **PA-R-2193** | 5'ATA GTA GCA TTG CCA CAA CTA 3' | 21 | **RLQP** |
| **HA Primers** | | | |
| **F1-6** | 5’ TAG CAA AAG CAG GGG AAT TTC TT 3’ | 23 | **RLQP** |
| H9- Rev | 5’ GCC ACC TTT TTC AGT CTG ACA TT 3’ | 23 | **Ben Shabat et al., 2011** |
| **H9-For** | 5’ GGAAGAATTAAT TATTATTGG TCG GTAC 3’ | 28 | **Ben Shabat et al., 2011** |
| **HT7R** | 5’ TAA TAC GAC TCA CTA TAA GTA CAA ACA AGG GTG 3’ | 33 | **SEPRL** |
| **NS Primers** | | | |
| **NS-8FV2** | **5**’AAA AGC AGG GTG ACA AAG ACA TAA **3’** | 24 | **FLI** |
| **NS-8RV2** | **5’**AGT AGA AAC AAG GGT GTT TTT TAT CA **3’** | 26 | **FLI** |
| **NS-R598** | **5**’GAG TTA TCA TTC CAT TCA AGT CC **3’** | 23 | **SEPRL** |
| **NS-F327** | **5**’GACTGG TTC ATG CTC ATG CC **3’** | 20 | **SEPRL** |

- Most of these primers were designed by Reference laboratory of quality control on poultry productions (RLQP), in Egypt.
- FLI:  Friedrich-Loeffler-Institute
