# Supplementary material for: Molecular pathogenic and host range determinants of reassortant Egyptian low pathogenic avian influenza H9N2 viruses from backyard chicken
Source: Int J Vet Sci Med. 2019 Jul 11;7(1):10–9. doi: 10.1080/23144599.2019.1637046 (PMC6776986; doi:10.1080/23144599.2019.1637046)
Supplement: Supplemental Material [file TVSM_A_1637046_SM9960.zip › Supplementary table 3.doc]

**Supplementary table 3: Accession no. of the sequenced genes HA, PB1, PB2, PA and NS of the isolates in this study:**

| **Isolate ID** | **HA** | **PB1** | **PB2** | **PA** | **NS** |
| --- | --- | --- | --- | --- | --- |
| A/Chicken/Egypt/ABD1/2015 | MG662416 | MG706976 | MG706981 | MG720150 | MG720155 |
| A/Chicken/Egypt/ABD2/2015 | MG662417 | MG706977 | MG706982 | MG720151 | MG720156 |
| A/Chicken/Egypt/ABD3/2015 | MG662418 | MG706978 | MG706983 | MG720152 | MG720157 |
| A/Chicken/Egypt/ABD4/2015 | MG662419 | MG706979 | MG706984 | MG720153 | MG720158 |
| A/Chicken/Egypt/ABD5/2015 | MG662420 | MG706980 | MG706985 | MG720154 | MG720159 |
